# Mutation of *GLR2* confers enhanced glufosinate resistance and salt tolerance in rice

**DOI:** 10.1093/plphys/kiae588

**Published:** 2024-11-05

**Authors:** Weimin Cheng, Yan Ren, Jiayi Wang, Chunpeng Chen, Cheng Fang, Lingling Peng, Dongyang Zhang, Liangzhi Tao, Yue Zhan, Kun Wu, Yuejin Wu, Binmei Liu, Xiangdong Fu, Yafeng Ye

**Affiliations:** Key Laboratory of High Magnetic Field and Ion Beam Physical Biology, Hefei Institutes of Physical Science, Chinese Academy of Sciences, Hefei 230031, China; Key Laboratory of High Magnetic Field and Ion Beam Physical Biology, Hefei Institutes of Physical Science, Chinese Academy of Sciences, Hefei 230031, China; Key Laboratory of High Magnetic Field and Ion Beam Physical Biology, Hefei Institutes of Physical Science, Chinese Academy of Sciences, Hefei 230031, China; Key Laboratory of High Magnetic Field and Ion Beam Physical Biology, Hefei Institutes of Physical Science, Chinese Academy of Sciences, Hefei 230031, China; Key Laboratory of High Magnetic Field and Ion Beam Physical Biology, Hefei Institutes of Physical Science, Chinese Academy of Sciences, Hefei 230031, China; Key Laboratory of High Magnetic Field and Ion Beam Physical Biology, Hefei Institutes of Physical Science, Chinese Academy of Sciences, Hefei 230031, China; Key Laboratory of High Magnetic Field and Ion Beam Physical Biology, Hefei Institutes of Physical Science, Chinese Academy of Sciences, Hefei 230031, China; Key Laboratory of High Magnetic Field and Ion Beam Physical Biology, Hefei Institutes of Physical Science, Chinese Academy of Sciences, Hefei 230031, China; Key Laboratory of High Magnetic Field and Ion Beam Physical Biology, Hefei Institutes of Physical Science, Chinese Academy of Sciences, Hefei 230031, China; New Cornerstone Science Laboratory, Institute of Genetics and Developmental Biology, Chinese Academy of Sciences, Beijing 100101, China; Key Laboratory of High Magnetic Field and Ion Beam Physical Biology, Hefei Institutes of Physical Science, Chinese Academy of Sciences, Hefei 230031, China; Key Laboratory of High Magnetic Field and Ion Beam Physical Biology, Hefei Institutes of Physical Science, Chinese Academy of Sciences, Hefei 230031, China; New Cornerstone Science Laboratory, Institute of Genetics and Developmental Biology, Chinese Academy of Sciences, Beijing 100101, China; College of Life Sciences, University of Chinese Academy of Sciences, Beijing 100049, China; Key Laboratory of High Magnetic Field and Ion Beam Physical Biology, Hefei Institutes of Physical Science, Chinese Academy of Sciences, Hefei 230031, China

## Abstract

The GLR2 transcription factor regulates glufosinate resistance and enhances abiotic stress tolerance in rice through interaction with the GLR1 transcription factor and modulation of gene expression.

Dear Editor,

Rice (*Oryza sativa* L.) is a staple crop feeding more than half of the world's population. However, rice production is often challenged by the presence of weeds, which substantially reduce crop yield and quality. Effective weed management is therefore essential, and herbicides like glufosinate have become widely used due to their nonselective, broad-spectrum weed control capabilities (Jin et al. 2022). Despite its effectiveness, the use of glufosinate in rice cultivation is limited because of its toxicity to the rice plant itself. Thus, developing glufosinate-resistant rice cultivars has been a focus of recent molecular breeding efforts.

Previous studies have demonstrated that enhancing the activity of endogenous glutamine synthetase (GS) and reducing the enzyme's sensitivity to glufosinate can confer resistance to this herbicide in plants (Zhang et al. 2022; Ren et al. 2023a, 2023b). It has been well documented that point mutations in endogenous GS genes can significantly improve plant resistance to glufosinate (Ren et al. 2023a, 2023b). Such mutations typically result in target-site resistance, where the altered GS becomes less susceptible to inhibition by the herbicide (Pornprom et al. 2009; Avila-Garcia et al. 2012). Consequently, GS genes, including *OsGS1;1*, have emerged as key targets for the development of glufosinate-resistant crops through gene editing technologies (Ren et al. 2023a, 2023b). Glufosinate resistant 2/auxin response factor 18 (GLR1/ARF18) and SQUAMOSA Promoter Binding Protein-Like 10 play a crucial role in the regulation of glufosinate resistance by directly controlling the expression of the *GS1* gene (Ren et al. 2023a, 2023b; Xia et al. 2023; Xia et al. 2024). Despite these advances, it remains to be fully elucidated whether additional mechanisms, beyond target-site resistance of GS, contribute to glufosinate resistance in plants.

Previously, we identified 2 glufosinate-resistant mutants, *glr1* and *glr2*, from the *japonica* cultivar Jingeng818 (wild type [WT]) through heavy ion beam mutagenesis and glufosinate screening. The *glr1* mutant and its associated molecular mechanisms have been reported (Ren et al. 2023a, 2023b). In this study, we further investigate the *glr2* mutant to uncover its underlying resistance mechanisms. To validate the glufosinate resistance of the *glr2* mutant, we treated seedlings with 500 g ai ha^−1^ of glufosinate. Compared with the WT, the *glr2* mutant showed significantly enhanced resistance, as evidenced by higher survival rates (Fig. 1, A and B). Glufosinate treatment led to better physiological performance in *glr2* compared with WT, including higher chlorophyll content (Fig. 1C), shoot height, root length, fresh weight, and dry weight (Supplementary Fig. S1, A to D). Given that glufosinate exerts its herbicidal effect by inhibiting GS activity, leading to ammonia accumulation and reactive oxygen species (ROS) production, we measured these parameters in WT and *glr2* plants. The *glr2* mutant exhibited significantly lower levels of ammonia, hydrogen peroxide (H_2_O_2_), and malondialdehyde (MDA) compared with WT after glufosinate treatment (Fig. 1, D to F). Additionally, GS, superoxide dismutase (SOD), and catalase (CAT) activities were notably higher in *glr2* (Fig. 1, G to I). Interestingly, while both WT and *glr2* plants showed increased ROS accumulation after glufosinate treatment, the ROS levels in *glr2* were significantly lower compared with the WT during the 4-d observation period (Supplementary Fig. S2), suggesting that *glr2* has a superior capacity to limit ROS accumulation.

**Figure 1.**
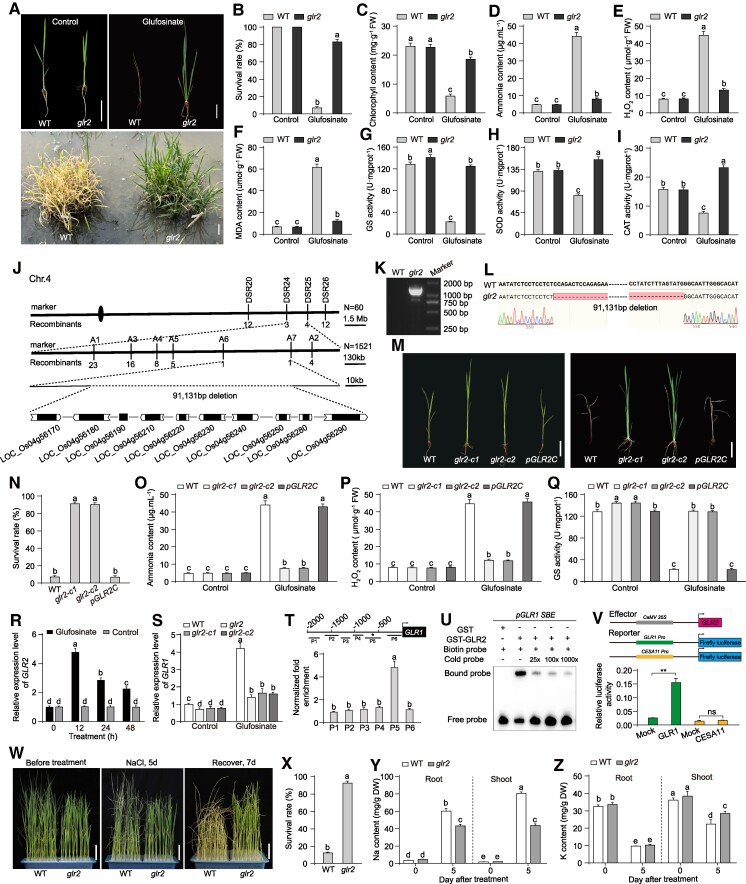
GLR2 regulates glufosinate resistance in rice. **A** to **I)** Glufosinate tolerance of WT and *glr2*. **A)** Fourteen-day-old seedlings were treated with 500 g ai ha^−1^ glufosinate for 2 wk. The control group refers to the seedlings before being treated with glufosinate. **B)** The survival rate, **C)** chlorophyll content, **D)** ammonia content, **E)** H_2_O_2_ content, **F)** MDA content, **G)** GS activity, **H)** SOD activity, and **I)** CAT activity of seedlings in **A)**. Data are means ± Sd (*n* = 10). **J)** Map-based cloning of *GLR2* gene. **K)** The amplified DNA fragments that cover the mutation site of WT and *glr2*. **L)** Sequencing chromatograms of the PCR-amplified DNA fragments shown in **K)**. **M** to **Q)** The glufosinate tolerance of the WT, 2 independent glr2-crispr lines (*glr2-c1* and *glr2-c2*), and complementation line *pGLR2C*. **M)** Fourteen-day-old seedlings were treated with 500 g ai ha^−1^ glufosinate for 2 wk. The control group refers to the seedlings before being treated with glufosinate. **N)** Survival rates, **O)** ammonia content, **P)** H_2_O_2_ content, and **Q)** GS activity of seedlings in **M)**. Data are means ± Sd (*n* = 10). **R)***GLR2* expression levels in 14-d-old seedlings treated with 500 g ai ha^−1^ glufosinate for indicated time. Data are means ± Sd (*n* = 3). **S)***GLR1* expression levels 14-d-old seedlings treated with 500 g ai ha^−1^ glufosinate after 48 h. Data are means ± Sd (*n* = 3). **T)** ChIP-quantitative PCR assays indicate that GLR2 binds to *GLR1* promoters in vivo. Data are means ± Sd (*n* = 3). **U)** EMSAs indicate GLR2 directly binds to GTAC motifs in *GLR1* promoters. **V)** Transactivation assays indicate GLR2 promoter *GLR1* transcription in rice protoplast. Data are means ± Sd (*n* = 3). **W** to **Z)** The *glr2* mutant exhibits significantly salt tolerance. **W)** Salt tolerance of WT and *glr2* plants. Fourteen-day-old seedlings were treated with 150 mm NaCl for 5 d, followed by recovery for 7 d under normal conditions. **X)** Survival rates of seedlings in **W)**. Data are means ± Sd (*n* = 3). Na^+^**Y)** and K^+^**Z)** contents in WT and *glr2* plants subjected to salt stress. Data are means ± Sd (*n* = 5). Each replicate contains 30 to 40 plants in **X)** and 10 plants in **Y** and **Z)**. Scale bars represent 5 cm in **W)**. Images in **A** and **M)** were digitally extracted for comparison. Scale bars represent 5 cm in **A** and **M)**. Different letters represent a significant difference at *P* < 0.05 determined by Tukey's honest significant difference test. The asterisks (**) indicate *P* < 0.01 from Student's *t*-tests.

To explore the molecular basis of glufosinate resistance in *glr2*, we performed map-based cloning and identified a 91-kb deletion on Chromosome 4, which includes the *GLR2* gene. Sequencing confirmed a 91,131-bp deletion (Fig. 1, J to L). We generated targeted knockouts of genes within this region and found that mutations in LOC_Os04g56170, corresponding to *GLR2*, conferred glufosinate resistance (Fig. 1, M to Q and Supplementary Figs. S3 to S13), confirming *GLR2* as the key gene responsible for the observed resistance phenotype. *GLR2* encodes an SQUAMOSA promoter binding protein-like (SPL) family transcription factor, SPL8, previously implicated in the regulation of rice leaf ligule and panicle development (Lee et al. 2007; Ishii et al. 2013; Zhu et al. 2013). Our expression analysis revealed that *GLR2* is expressed in various tissues, with higher levels in leaves and panicles (Supplementary Fig. S14A), and its expression is induced by glufosinate treatment (Fig. 1R). As a transcription factor, GLR2 is nuclear localized and possesses strong transcriptional activation activity (Supplementary Fig. S14, B and C).

Given the similar resistance profiles of *glr1* and *glr2*, we hypothesized that GLR2 might regulate *GLR1* expression. Indeed, GLR1 transcript levels were significantly reduced in the *glr2* mutant under both control and glufosinate-treated conditions (Fig. 1S). SPL family transcription factors typically bind to GTAC motifs in target gene promoters. We identified 3 GTAC motifs in the 2-kb promoter region of *GLR1* (Supplementary Fig. S15), and chromatin immunoprecipitation (ChIP) assays confirmed that GLR2 directly binds to these motifs (Fig. 1T). This interaction was further validated by electrophoretic mobility shift assay (EMSA) (Fig. 1U), and reporter assays demonstrated that GLR2 enhances GLR1 promoter activity (Fig. 1V). These findings suggest that GLR2 directly binds to the *GLR1* promoter and upregulates its expression. In WT plants, glufosinate treatment induces *GLR2* expression, which in turn promotes *GLR1* expression, leading to the suppression of downstream genes involved in ammonia and ROS detoxification (e.g. *GS1*, *CYP51G3*, and *CATA*; Ren et al. 2023a, 2023b). However, in the *glr2* mutant, the absence of GLR2 results in reduced *GLR1* expression (Fig. 1S), allowing the activation of these detoxification pathways and conferring resistance to glufosinate.

Moreover, GLR2 and GLR1 were found to interact at the protein level, as demonstrated by luciferase complementation imaging, co-immunoprecipitation, and bimolecular fluorescence complementation assays, showing that these proteins interact within the nucleus (Supplementary Fig. S16). Additionally, GLR2 also binds to the *GS1* promoter and regulates its expression, and this interaction is enhanced by GLR1, further repressing *GS1* expression (Supplementary Fig. S17). These results indicate that GLR2 not only regulates *GLR1* transcription but also collaborates with GLR1 to modulate downstream gene expression.

In addition to its role in glufosinate resistance, the *glr1* mutant has also been shown to exhibit significant tolerance to salt stress (Ren et al. 2023a, 2023b). Given the similarities in the resistance mechanisms between *glr1* and *glr2*, it is plausible to hypothesize that the mutation of *GLR2* might also possess enhanced salt tolerance. Interestingly, both the *glr2* mutant, as well as the *glr2-c1* and *glr2-c2* lines, also exhibited enhanced tolerance to salinity (Fig. 1W and Supplementary Fig. S18). Salt stress experiments demonstrated that *glr2* plants had higher survival rates and better ion homeostasis compared with WT, as indicated by Na^+^ and K^+^ contents in both shoots and roots (Fig. 1, X to Z). Our findings suggest that manipulating the GLR2/GLR1 regulatory module could be a promising strategy for developing rice varieties with enhanced resistance to both herbicides and environmental stresses, thereby contributing to more sustainable agricultural practices.

## Supplementary Material

kiae588_Supplementary_Data

## Data Availability

The data underlying this article are available in the article and in its online supplementary material.

## Supplementary data

The following materials are available in the online version of this article.

**Supplementary Materials and Methods.**

**Supplementary Figure S1.** Glufosinate tolerance of WT and *glr2*.

**Supplementary Figure S2.** H_2_O_2_ accumulation process after glufosinate treament.

**Supplementary Figure S3.** Generation and characterization of clustered regularly interspaced short palindromic repeats-CRISPR associated protein 9 (CRISPR-Cas9)–induced mutations in the *GLR2/SPL8* (LOC_Os04g56170) gene.

**Supplementary Figure S4.** Generation and analysis of LOC_Os04g56180 (A1) gene mutant using CRISPR-Cas9.

**Supplementary Figure S5.** Generation and analysis of LOC_Os04g56190 (A2) gene mutant using CRISPR-Cas9.

**Supplementary Figure S6.** Generation and analysis of LOC_Os04g56210 (A3) gene mutant using CRISPR-Cas9.

**Supplementary Figure S7.** Generation and analysis of LOC_Os04g56220 (A4) gene mutant using CRISPR-Cas9.

**Supplementary Figure S8.** Generation and analysis of LOC_Os04g56230 (A5) gene mutant using CRISPR-Cas9.

**Supplementary Figure S9.** Generation and analysis of LOC_Os04g56240 (A6) gene mutant using CRISPR-Cas9.

**Supplementary Figure S10.** Generation and analysis of LOC_Os04g56250 (A7) gene mutant using CRISPR-Cas9.

**Supplementary Figure S11.** Generation and analysis of LOC_Os04g56280 (A8) gene mutant using CRISPR-Cas9.

**Supplementary Figure S12.** Generation and analysis of LOC_Os04g56290 (A9) gene mutant using CRISPR-Cas9.

**Supplementary Figure S13.** Phenotypic and physiological analysis of WT, *glr2* mutant lines (*glr2-c1* and *glr2-c2*), and the *GLR2*-complemented line (*pGLR2C*) under control and glufosinate-treated conditions.

**Supplementary Figure S14.** Expression patterns, subcellular localization, and transcriptional activity of GLR2.

**Supplementary Figure S15.** Sequence analysis of the promoter region of the *GLR1* gene, highlighting SPL-binding elements.

**Supplementary Figure S16.** Interaction between GLR1 and GLR2 proteins.

**Supplementary Figure S17.** Regulation of *GS1* expression by GLR1 and GLR2 in rice.

**Supplementary Figure S18.** Salt tolerance of *glr2-c1* and *glr2-c2* mutants compared with WT.

**Supplementary Table S1.** Primers used in this study.

## Dive Curated Terms

The following phenotypic, genotypic, and functional terms are of significance to the work described in this paper:

GLR2 Gramene: LOC_Os04g56170
GLR2 Araport: LOC_Os04g56170

GS1 Gramene: LOC_Os02g50240
GS1 Araport: LOC_Os02g50240

GLR1 Gramene: LOC_Os06g47150
GLR1 Araport: LOC_Os06g47150

SPL8 Gramene: LOC_Os04g56170
SPL8 Araport: LOC_Os04g56170
