# Supplementary material for: Mutation of GLR2 confers enhanced glufosinate resistance and salt tolerance in rice
Source: Plant Physiol. 2024 Nov 5;197(1):kiae588. doi: 10.1093/plphys/kiae588 (PMC11663548; doi:10.1093/plphys/kiae588)
Supplement: kiae588_Supplementary_Data [file kiae588_supplementary_data.zip › Supplementary Data.pdf]

Mutation of *GLR2* confers enhanced glufosinate resistance and salt tolerance in rice

Weimin Cheng[1], Yan Ren[1], Jiayi Wang[1], Chunpeng Chen[1], Cheng Fang[1], Lingling Peng[1], Dongyang Zhang[1], Liangzhi Tao[1], Yue Zhan[1], Kun Wu[2], Yuejin Wu[1], Binmei Liu[1, *], Xiangdong Fu[2, *], Yafeng Ye[1, *]

[1]Key Laboratory of High Magnetic Field and Ion Beam Physical Biology, Hefei Institutes of Physical Science, Chinese Academy of Sciences, Hefei 230031, China

[2]New Cornerstone Science Laboratory, Institute of Genetics and Developmental Biology, Chinese Academy of Sciences, Beijing 100101, China

[3]College of Life Sciences, University of Chinese Academy of Sciences, Beijing 100049, China

[*]Correspondence should be addressed to Y.Y. (yyfeng86@163.com), X.F. (xdfu@genetics.ac.cn) or B.L. (liubm@ipp.ac.cn)

Supplemental Materials & Methods

Plant materials and growing conditions.

Rice (*Oryza sativa*) seedlings were grown hydroponically in a modified Kimura B solution as described previously (Deng et al., 2022). The solution contained 0.09 mM K_2SO_4, 0.5 mM $(NH_4)_2SO_4$, 0.54 mM $MgSO_4 \cdot 7H_2O$, 1 mM KNO_3, 0.3 mM $CaCl_2$, 46.2 μM $Na_2MoO_4 \cdot 2H_2O$, 0.18 mM NaH_2PO_4, 0.32 μM $CuSO_4 \cdot 5H_2O$, 16 μM $Na_2SiO_3 \cdot 9H_2O$, 9.14 μM $MnCl_2 \cdot 4H_2O$, 0.76 μM $ZnSO_4 \cdot 7H_2O$, and 40 μM Fe(II)-EDTA (pH 5.8). The nutrient solution was renewed every 5 days. Seedlings were cultivated in a growth chamber maintained at 28 °C, with a 16-hour light/8-hour dark photoperiod, a light intensity of ~200 μmol photons m^{-2} s^{-1}, and approximately 70% humidity.

Ammonia accumulation

Ammonia accumulation assays were carried out following a modified protocol as described previously (Dayan et al., 2017). Seedlings were grown in a greenhouse as previously described. At the 4-leaf growth stage, the seedlings were treated with glufosinate at a rate of 500 g ai ha^{-1} for the specified time intervals. Leaf discs, 5 mm in diameter, were collected and placed in wells containing 100 μL of water. The plates were then rapidly frozen at −80 °C, and cell membranes were disrupted through two cycles of freezing and thawing. Subsequently, 50 μL from each well was transferred to a new plate. To measure ammonia levels, 150 μL of water, 100 μL of phenol nitroprusside solution, and 50 μL of alkaline hypochlorite solution were added to each sample. After a 30-minute incubation at room temperature, absorbance was recorded at 540 nm. Each experiment was replicated three times, using ten seedlings for each time point.

H_2O_2 (Hydrogen peroxide) staining Assays

DAB staining for H_2O_2 accumulation was performed following the protocol

outlined by Takano et al. (Takano et al., 2019), with slight modifications. Briefly, leaf samples were incubated in DAB staining solution at 28°C in the dark for 24 hours. After staining, the leaf discs were thoroughly washed with distilled water and then boiled in 70% (v/v) ethanol, with the ethanol solution being refreshed every 20 minutes for a total of four cycles. The discs were subsequently stored in 70% (v/v) ethanol for 12 hours before being scanned.

Determination of various antioxidant indexes

The levels of H_2O_2, MDA, GS, SOD, and CAT were measured as described previously (Zhang et al., 2019), with some modifications. At the 4-leaf growth stage, seedlings were treated with 500 g ai ha^{-1} of glufosinate for the specified durations. Leaves were collected and immediately stored at -80°C until further analysis. The samples were then ground in liquid nitrogen. The antioxidant indices were measured using assay kits (Solarbio, China).

Chlorophyll content measurement

Rice seedlings were incubated in 80% acetone and vigorously shaken in the dark for 24 hours at room temperature. Afterward, the samples were centrifuged at 10,000 g for 10 minutes, and the supernatant was collected for chlorophyll measurement. Absorbance was measured at 663 nm and 645 nm. Chlorophyll content was then calculated following the method described previously (Wang et al., 2015a).

Mapping of *glr2* locus

To analyze the inheritance pattern, the glufosinate-tolerant mutant *glr2* was crossed with its wild-type counterpart. Phenotypic data from the F2 generation were analyzed using a chi-square (χ^2) test to assess the goodness of fit with the expected Mendelian segregation ratio (Fisher, 1936). Using 60 F_2 mutant individuals, the *glr2* locus was mapped to chromosome 4, between the InDel

markers DSR24 and DSR25. Fine-mapping, based on 1521 F_2 mutant individuals, further narrowed the mutant locus to an approximately 230-kb region between the dCAPS markers A6 and A7. Chromosome walking and sequencing analysis were then employed to confirm the fragment deletion mutation type in the *glr2* mutant. The primers used for map-based cloning and identification of the mutation site are listed in Supplementary Table S1.

Plasmid construction and generation of transgenic plants

For the complementation of the *glr2* mutant, the full-length *GLR2* coding sequence, along with a 2.5 kb upstream region from the start codon and a 0.2 kb downstream region from the termination codon, was cloned into the *pCAMBIA2300* vector to generate the *pGLR2::GLR2* (*pGLR2C*) construct. To create corresponding gene deletions in the *glr2* mutant, the CRISPR/Cas9 system was employed. The construction of CRISPR/Cas9 binary vectors followed the method as previously described (Ma *et al.*, 2015). The Cas9 plant expression vector and sgRNA expression vector were kindly provided by Prof. Yao-Guang Liu. All transgenic rice plants were generated through Agrobacterium-mediated transformation as previously described (Shri *et al.*, 2013).

Subcellular Localization Assay

To observe the subcellular localization of GLR2, green fluorescent protein (GFP) was fused to the C-terminus of GLR2 and inserted into the pCAMBIA2300 vector under the control of the cauliflower mosaic virus (CaMV) 35S promoter. The GLR2-GFP construct, along with GLR1 fused to red fluorescent protein (RFP), was co-expressed in *Nicotiana benthamiana* leaves through Agrobacterium-mediated infiltration. After 48 hours, the fluorescence of GLR2-GFP and GLR1-RFP in the leaf tissues was examined using a confocal laser scanning microscope (Zeiss LSM980) to determine their subcellular localization and co-localization. GFP signals were recorded using a white light

laser with an excitation wavelength of 488 nm and an emission range of 510–550 nm. Red signals were recorded with an excitation wavelength of 555 nm and an emission range of 575–630 nm.

Transcriptional Activity and Transient Expression Regulation Assays

The coding sequence (CDS) of *GLR2* was amplified using the Donor-GLR2-F and Donor-GLR2-R primers, with cDNA synthesized from JG818 leaf tissue as the template. The amplified product was first inserted into an intermediate donor vector and confirmed by sequencing. The verified product was then transferred into the *p35S::BD* vector using Gateway recombination cloning technology, following the manufacturer’s instructions (Invitrogen, Carlsbad, CA, USA; 11789100 and 11791100), to generate the *p35S::BD-GLR2* plasmid. Meanwhile, the promoter fragment of the target gene was ligated into the 5×GAL4-LUC vector, resulting in a promoter-LUC construct specific to the target gene. The empty GAL4BD vector served as a negative control. A pTRL plasmid containing the Renilla luciferase (LUC) gene driven by the 35S promoter was used as an internal control. The transactivation assay was conducted as described previously (Wang et al., 2015b). The *pTRL*, effector, and reporter constructs were co-transformed into a rice protoplast system and incubated in the dark overnight. Luciferase activity was measured as previously described (Liu et al., 2018).

RT-qPCR analysis

RT-qPCR analysis assays were conducted using the method described by Li (Li et al., 2018). Total RNA was extracted from various seedling organs using TRIzol reagent (Invitrogen). The RNA samples were then treated with RNase-free DNase I (Invitrogen) according to the manufacturer's protocol to remove any contaminating DNA. Full-length cDNA was synthesized using a cDNA synthesis kit (GENESTAR). Transcript levels were evaluated by qPCR using the manufacturer’s instructions (GENESTAR), with three independent RNA

preparations serving as biological replicates. Gene expression levels were normalized to *OsActin1* as the internal control. The primers used for RT-qPCR are listed in Supplementary Table S1.

ChIP–qPCR assay

ChIP assays were performed following the protocol outlined previously (O'Geen et al., 2010), with some modifications. Two-week-old transgenic rice seedlings expressing *pActin::3*myc-GLR2* were ground in liquid nitrogen and crosslinked with 1% formaldehyde under vacuum to fix the protein-DNA interactions. Nuclei were then isolated and lysed, and the chromatin was sheared by ultrasonication into fragments ranging from 200 to 500 bp. The supernatant was pre-cleared with protein A agarose beads pre-absorbed with sheared salmon sperm DNA. A portion of the supernatant was reserved as the input control. Immunoprecipitation was carried out using an anti-myc antibody (1:3,000, ABclonal) at 4°C, while a mock sample was prepared using an equal volume of supernatant without the antibody. After reverse crosslinking, the resulting DNA was used as a template for qPCR. The specific primer sequences utilized for qPCR are provided in Supplementary Table S1.

EMSA

The coding sequences of *GLR2* were cloned into the *pGEX-4T-1* vector (GE Healthcare) using Gateway recombination technology as above described. The resulting GST-tagged proteins were purified using Glutathione Sepharose 4B (GE Healthcare, 17-0756) according to the manufacturer’s protocol. DNA probes were generated and labeled with biotin using a biotin labeling kit (Invitrogen). Electrophoretic mobility shift assays (EMSAs) were performed using the LightShift Chemiluminescent EMSA Kit (Thermo Fisher Scientific, 20148), following the procedures outlined by (Wang et al, 2015c). The primers used for amplifying the DNA probes are listed in Supplementary Table S1.

Co-IP assays

Full-length cDNAs of GLR2 and GLR1 were amplified and subsequently cloned into the PUC-35S-Flag-RBS or PUC-35S-HA-RBS vectors. Rice protoplasts were transfected with 100 μg of plasmid DNA and incubated overnight under low light conditions. Total protein was extracted from the harvested protoplasts using a lysis buffer containing 50 mM HEPES (pH 7.5), 150 mM KCl, 1 mM EDTA, 0.5% Triton-X 100, 1 mM DTT, and a protease inhibitor cocktail (Roche Life Science). The lysates were then incubated with Flag M2 Affinity Gel (Sigma-Aldrich, A2220) at 4°C for at least 4 hours, followed by 5–6 washes with extraction buffer. The bound proteins were eluted using 3×Flag peptide (Sigma-Aldrich, F4709). Immunoprecipitates were separated by SDS-PAGE and transferred onto a nitrocellulose membrane (GE Healthcare). The membranes were then probed with anti-HA (1:5,000, MBL, M180-7) or anti-DDDDK-tag mAb-HRP-DirectT antibodies (1:10,000, MBL, M185-7) to detect the proteins.

LCI assays

LCI assays were conducted following the method described previously with some modifications (Liu et al, 2018). Full-length cDNAs of *GLR2* and *GLR1* were amplified and cloned into pCAMBIA1300-cLUC and pCAMBIA1300-nLUC vectors, generating pCAMBIA-GLR1-cLuc and pCAMBIA-GLR2-nLUC constructs. These constructs, along with the p19 silencing plasmid, were co-transfected into *Nicotiana benthamiana* leaves via *Agrobacterium tumefaciens* infiltration. LUC activity was assessed according to established protocols. Approximately 36-48 hours post-infiltration, the transfected leaves were detached and sprayed with 1 mM luciferin (Promega, E1605). Luminescence signals were captured using a cooled CCD imaging system (Berthold, LB985).

BiFC assays

The CDS of GLR2 was cloned into p2YC, and RGB1 was cloned into the p2YN,

forming the GLR2-YFPc, GLR1-YFPn fusion proteins. The recombinant BiFC vectors were then co-transformed into *N. benthamiana* leaves. Fluorescence was observed using a LMS980 confocal laser-scanning microscope (Carl Zeiss, Jena, Germany) after 48 h. YFP signals were recorded with an emission range of 514 nm and an emission range of 527-550 nm.

Measurements of ion contents

Ion concentration measurements in rice tissues were conducted following the method as previously described (Ye et al., 2021). Tissue samples were initially heated at 105°C for 1 hour, then dried at 65°C until a constant weight was achieved. The dried samples were ground into a fine powder and digested with ultrapure nitric acid for 24 hours. The digested samples were subsequently boiled at 95°C for 10 minutes, repeated three times. Ion content was measured using an inductively coupled plasma optical emission spectrometer (ICP-MS, Agilent 7700 series, USA).

References

Avila-Garcia, W.V., Sanchez-Olguin, E., Hulting, A.G. and Mallory-Smith, C. (2012) Target-site mutation associated with glufosinate resistance in Italian ryegrass (Lolium perenne L. ssp. multiflorum). *Pest Manag Sci* **68**, 1248-1254.

Dayan, F.E., Owens, D.K., Corniani, N., Silva, F.M.L., Watson, S.B., Howell, J.L. and Shaner, D.L. (2017) Biochemical Markers and Enzyme Assays for Herbicide Mode of Action and Resistance Studies. *Weed Science* **63**, 23-63.

Deng, P., Jing, W., Cao, C., Sun, M., Chi, W., Zhao, S., Dai, J., Shi, X., Wu, Q., Zhang, B., Jin, Z., Guo, C., Tian, Q., Shen, L., Yu, J., Jiang, L., Wang, C., Chin, J.H., Yuan, J., Zhang, Q. and Zhang, W. (2022) Transcriptional repressor RST1 controls salt tolerance and grain yield in rice by regulating gene expression of asparagine synthetase. *Proc Natl Acad*

Sci U S A **119**, e2210338119.

Ye Y, Wang S, Wu K, Ren Y, Jiang H, Chen J, Tao L, Fu X, Liu B, Wu Y (2021) A Semi-Dominant Mutation in OsCESA9 Improves Salt Tolerance and Favors Field Straw Decay Traits by Altering Cell Wall Properties in Rice. *Rice* (N Y) **14**: 19.

Li, S., Tian, Y., Wu, K., Ye, Y., Yu, J., Zhang, J., Liu, Q., Hu, M., Li, H., Tong, Y., Harberd, N.P. and Fu, X. (2018) Modulating plant growth-metabolism coordination for sustainable agriculture. *Nature* **560**, 595-600.

Liu, Q., Han, R., Wu, K., Zhang, J., Ye, Y., Wang, S., Chen, J., Pan, Y., Li, Q., Xu, X., Zhou, J., Tao, D., Wu, Y. and Fu, X. (2018) G-protein betagamma subunits determine grain size through interaction with MADS-domain transcription factors in rice. *Nat Commun* **9**, 852.

Love, M.I., Huber, W. and Anders, S. (2014) Moderated estimation of fold change and dispersion for RNA-seq data with DESeq2. *Genome Biol* **15**, 550.

Ma, X., Zhang, Q., Zhu, Q., Liu, W., Chen, Y., Qiu, R., Wang, B., Yang, Z., Li, H., Lin, Y., Xie, Y., Shen, R., Chen, S., Wang, Z., Chen, Y., Guo, J., Chen, L., Zhao, X., Dong, Z. and Liu, Y.G. (2015) A Robust CRISPR/Cas9 System for Convenient, High-Efficiency Multiplex Genome Editing in Monocot and Dicot Plants. *Mol Plant* **8**, 1274-1284.

O'Geen, H., Frietze, S. and Farnham, P.J. (2010) Using ChIP-seq technology to identify targets of zinc finger transcription factors. *Methods Mol Biol* **649**, 437-455.

Shri, M., Rai, A., Verma, P.K., Misra, P., Dubey, S., Kumar, S., Verma, S., Gautam, N., Tripathi, R.D., Trivedi, P.K. and Chakrabarty, D. (2013) An improved Agrobacterium-mediated transformation of recalcitrant indica rice (Oryza sativa L.) cultivars. *Protoplasma* **250**, 631-636.

Takano, H.K., Beffa, R., Preston, C., Westra, P. and Dayan, F.E. (2019) Reactive oxygen species trigger the fast action of glufosinate. *Planta* **249**, 1837-1849.

Wang, R., Jing, W., Xiao, L., Jin, Y., Shen, L. and Zhang, W. (2015a) The Rice High-Affinity Potassium Transporter1;1 Is Involved in Salt Tolerance and Regulated by an MYB-Type Transcription Factor. *Plant Physiol* **168**, 1076-1090.

Wang, S., Li, S., Liu, Q., Wu, K., Zhang, J., Wang, S., Wang, Y., Chen, X., Zhang, Y., Gao, C., Wang, F., Huang, H. and Fu, X. (2015b) The OsSPL16-GW7 regulatory module determines grain shape and simultaneously improves rice yield and grain quality. *Nat Genet* **47**, 949-954.

Wang, Y., Xiong, G., Hu, J., Jiang, L., Yu, H., Xu, J., Fang, Y., Zeng, L., Xu, E., Xu, J., Ye, W., Meng, X., Liu, R., Chen, H., Jing, Y., Wang, Y., Zhu, X., Li, J. and Qian, Q. (2015c) Copy number variation at the GL7 locus contributes to grain size diversity in rice. *Nat Genet* **47**, 944-948.

Zhang, W., Zhu, K., Wang, Z., Zhang, H., Gu, J., Liu, L., Yang, J. and Zhang, J. (2019) Brassinosteroids function in spikelet differentiation and degeneration in rice. *J Integr Plant Biol* **61**, 943-963.

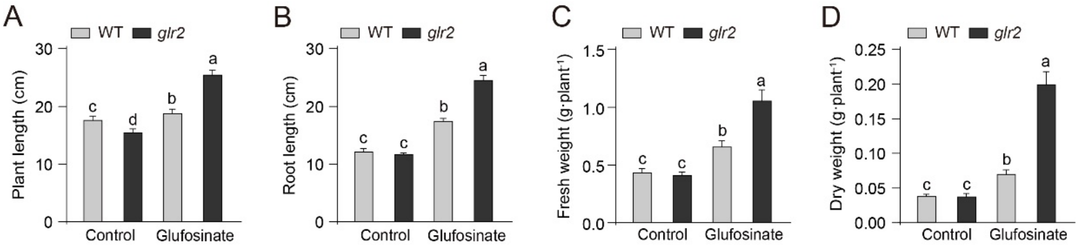

Supplementary Figure S1. Glufosinate tolerance of WT and *glr2*.

Fourteen-day-old seedlings were treated with 500g ai ha^{-1} glufosinate for two weeks. The control group refers to the seedlings before being treated with glufosinate. The plant length(A), root length(B), fresh weight(C), and dry weight(D) of seedlings of WT and *glr2*. Data are means ± SD (n = 10). The experiment was repeated three times. According to Tukey's honest significant difference (HSD) test, different letters indicate a significant difference at $P < 0.05$.

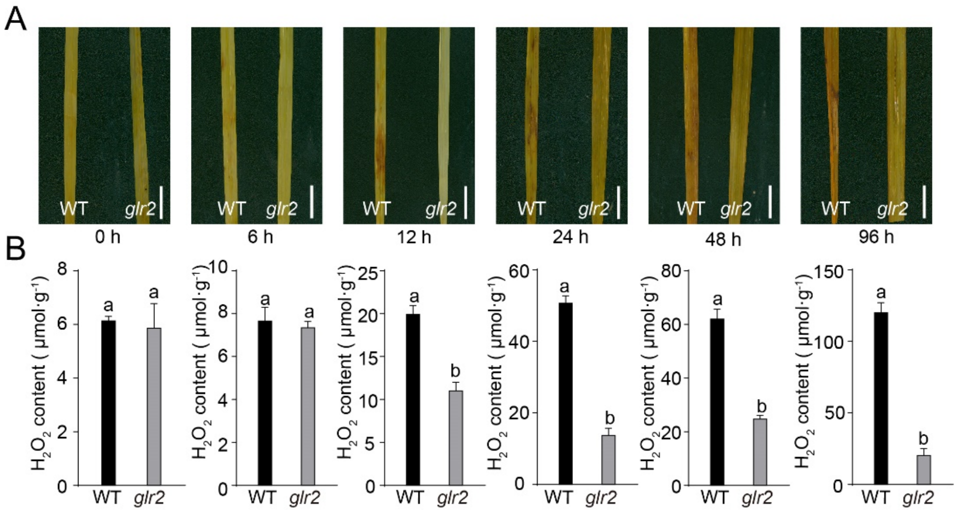

Supplementary Figure S2. H_2O_2 accumulation process after glufosinate treament. (A) Visual representation of H_2O_2 accumulation over time, showing leaf discoloration in WT and *glr2* plants at 0, 6, 12, 24, 48, and 96 hours post-treatment. (B) Quantitative analysis of H_2O_2 content in WT and *glr2* plants at the same time points. The scale bars in the figure represent 1 cm in (A). Data are means ± SD (n = 10). Different letters represent a significant difference at $P < 0.05$ determined by Tukey's honest significant difference (HSD) test.

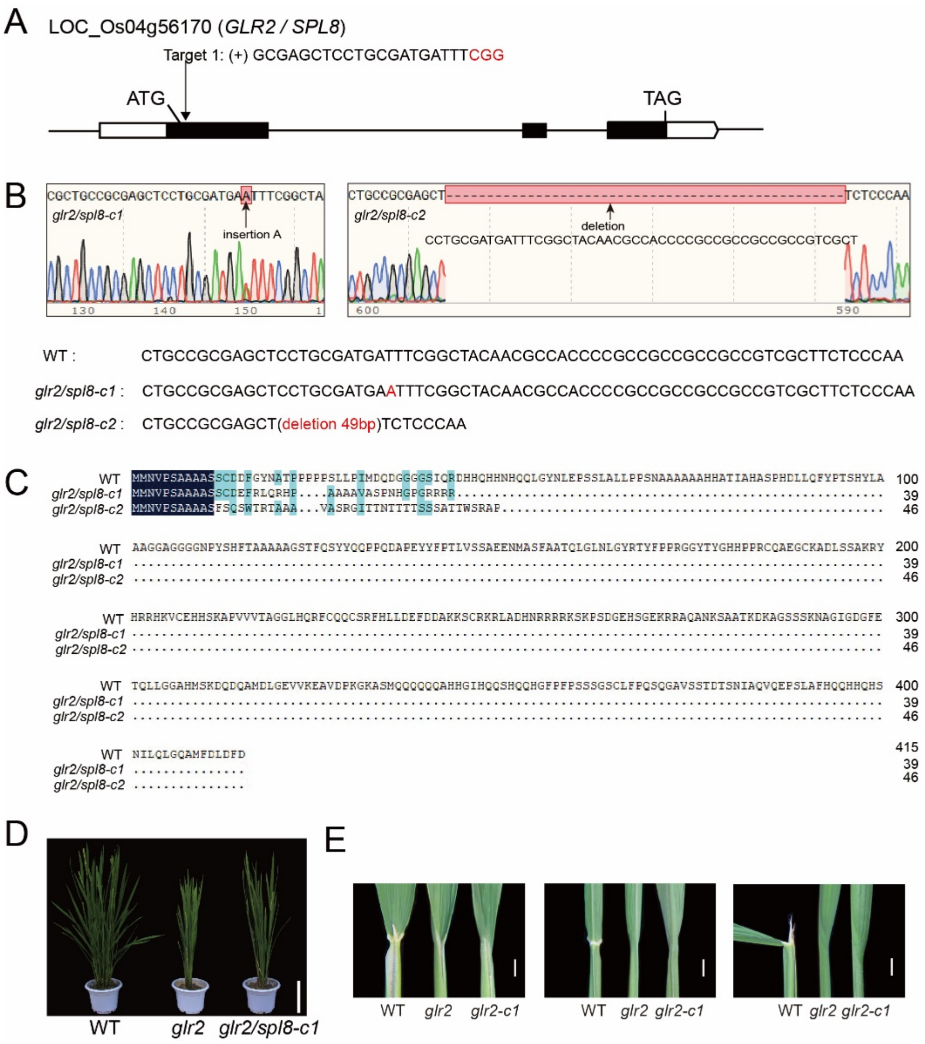

Supplementary Figure S3. Generation and characterization of CRISPR-Cas9-induced mutations in the *GLR2/SPL8* (LOC_Os04g56170) gene. (A) Schematic representation of the *GLR2* gene structure, with the CRISPR-Cas9 target site indicated. The target sequence and PAM are highlighted in red. (B) Sequencing chromatograms showing mutations in the *glr2/spl8-c1* and *glr2/spl8-c2* mutants. The *glr2/spl8-c1* mutant contains an A insertion, while the *glr2/sp8-c2* mutant has a 49 bp deletion. (C) Protein sequence alignment of the WT and mutant alleles, showing the impact of the mutations on the *GLR2* protein sequence. (D) Phenotypic comparison of WT, *glr2*, and *glr2/spl8-c1* plants. (E) Close-up images of the leaf angles in WT, *glr2*, and *glr2/spl8-c1* plants. Images were digitally extracted for comparison in (D) and (E). Scale bars in (D) and (E) = 5 cm and 1 cm, respectively.

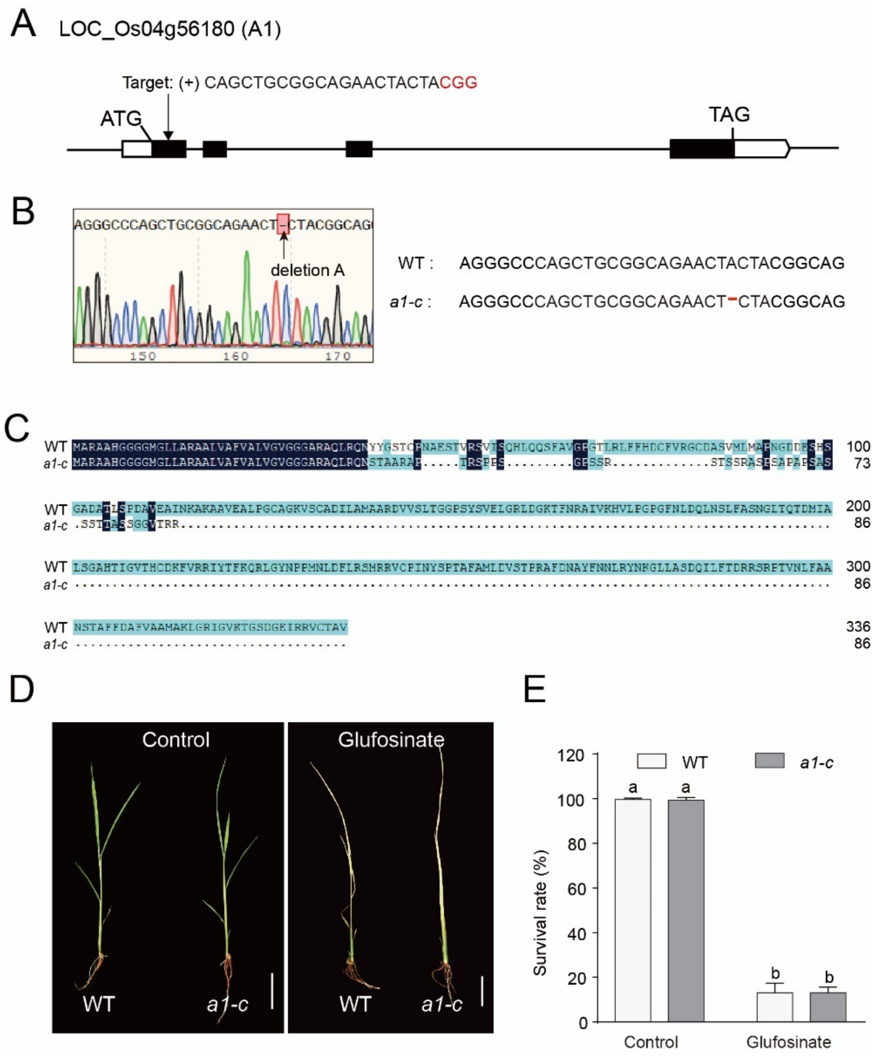

Supplementary Figure S4. Generation and analysis of LOC_Os04g56180 (A1) gene mutant using CRISPR-Cas9.

(A) Schematic representation of the LOC_Os04g56180 (A1) gene structure and the CRISPR-Cas9 target site. The target sequence and PAM are highlighted in red. (B) Sequencing chromatogram showing a single nucleotide deletion (deletion A) in the *a1-c* mutant. The WT sequence is shown for comparison. (C) Protein sequence alignment of WT and *a1-c*, showing the impact of the deletion on the amino acid sequence. (D) Phenotypic comparison of WT and *a1-c* plants under control and glufosinate-treated conditions. (E) Quantitative analysis of survival rates for WT and *a1-c* plants under control and glufosinate-treated conditions. Images were digitally extracted for comparison in (D) The scale bars in the figure represent 1 cm in (D). Data are means ± SD (n = 10). Different letters represent a significant difference at $P < 0.05$ determined by Tukey's honest significant difference (HSD) test.

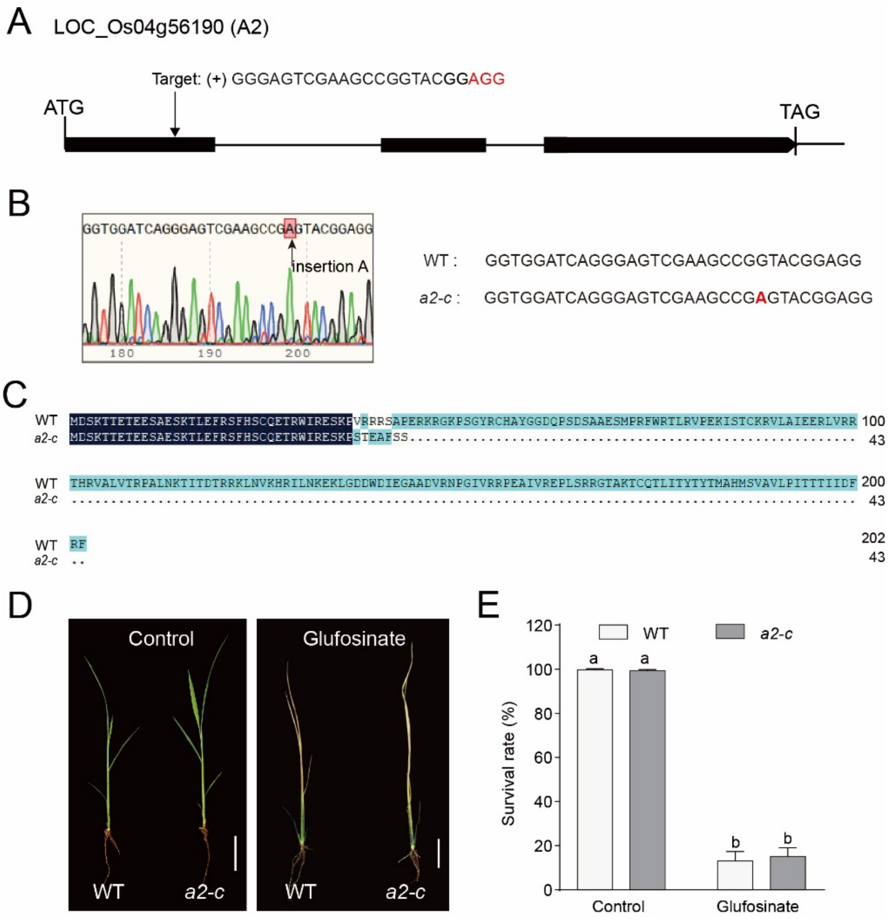

Supplementary Figure S5. Generation and analysis of LOC_Os04g56190 (A2) gene mutant using CRISPR-Cas9.

(A) Schematic representation of the LOC_Os04g56190 (A2) gene structure and the CRISPR-Cas9 target site. The target sequence and PAM are highlighted in red. (B) Sequencing chromatogram showing a single nucleotide insertion (insertion A) in the *a2-c* mutant. The WT sequence is shown for comparison. (C) Protein sequence alignment of WT and *a2-c*, showing the impact of the deletion on the amino acid sequence. (D) Phenotypic comparison of WT and *a2-c* plants under control and glufosinate-treated conditions. (E) Quantitative analysis of survival rates for WT and *a2-c* plants under control and glufosinate-treated conditions. Images were digitally extracted for comparison in (D). The scale bars in the figure represent 1 cm in (D). Data are means ± SD (n = 10). Different letters represent a significant difference at $P < 0.05$ determined by Tukey's honest significant difference (HSD) test.

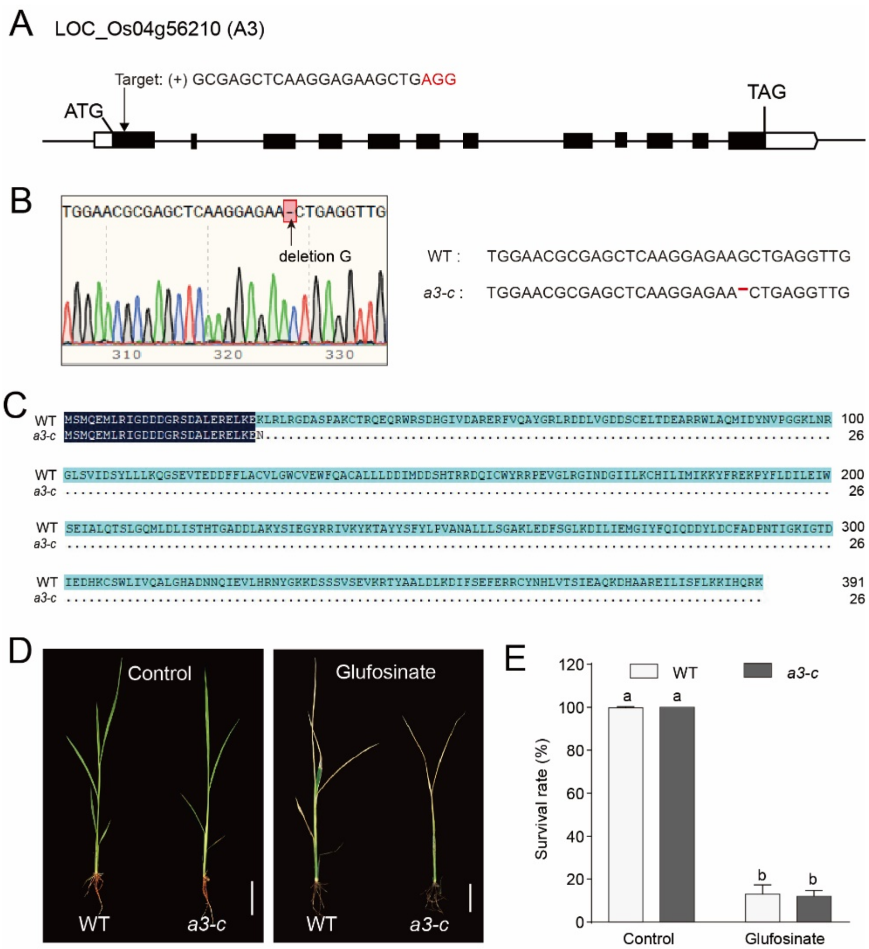

Supplementary Figure S6. Generation and analysis of LOC_Os04g56210 (A3) gene mutant using CRISPR-Cas9.

(A) Schematic representation of the LOC_Os04g56210 (A3) gene structure and the CRISPR-Cas9 target site. The target sequence and PAM are highlighted in red. (B) Sequencing chromatogram showing a single nucleotide deletion (deletion G) in the *a3-c* mutant. The WT sequence is shown for comparison. (C) Protein sequence alignment of WT and *a3-c*, showing the impact of the deletion on the amino acid sequence. (D) Phenotypic comparison of WT and *a3-c* plants under control and glufosinate-treated conditions. (E) Quantitative analysis of survival rates for WT and *a3-c* plants under control and glufosinate-treated conditions. Images were digitally extracted for comparison in (D). The scale bars in the figure represent 1 cm in (D). Data are means ± SD (n = 10). Different letters represent a significant difference at *P* < 0.05 determined by Tukey's honest significant difference (HSD) test.

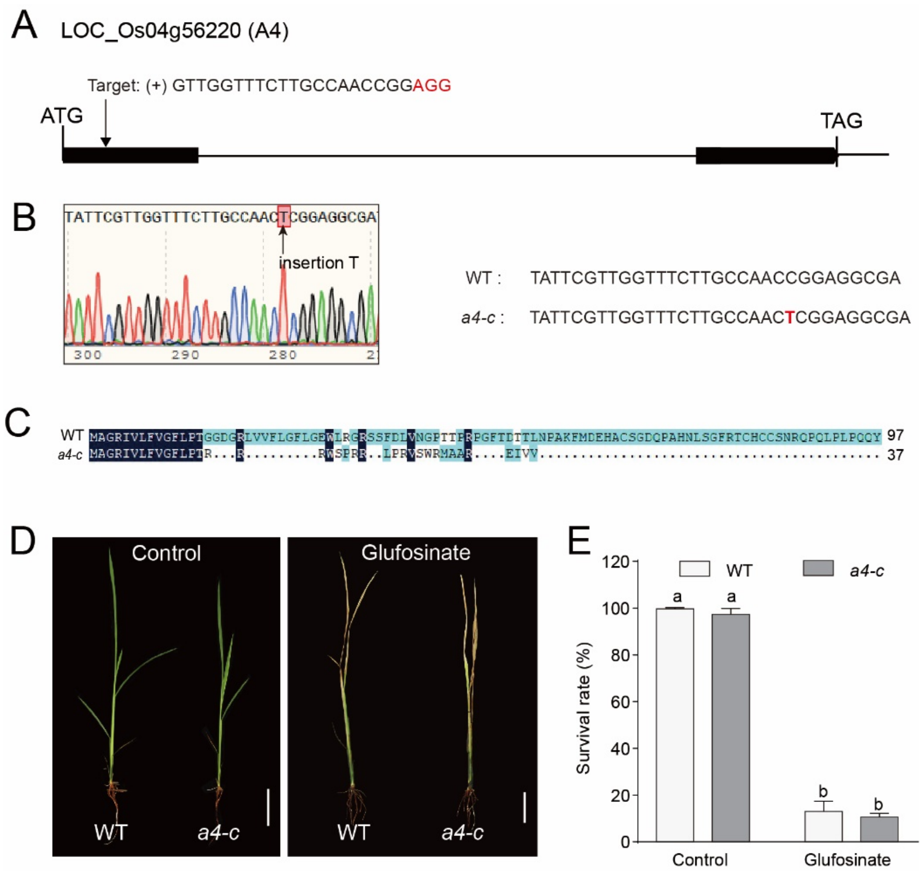

Supplementary Figure S7. Generation and analysis of LOC_Os04g56220 (A4) gene mutant using CRISPR-Cas9.

(A) Schematic representation of the LOC_Os04g56220 (A4) gene structure and the CRISPR-Cas9 target site. The target sequence and PAM are highlighted in red. (B) Sequencing chromatogram showing a single nucleotide insertion (insertion T) in the *a4-c* mutant. The WT sequence is shown for comparison. (C) Protein sequence alignment of WT and *a4-c*, showing the impact of the deletion on the amino acid sequence. (D) Phenotypic comparison of WT and *a4-c* plants under control and glufosinate-treated conditions. (E) Quantitative analysis of survival rates for WT and *a4-c* plants under control and glufosinate-treated conditions. Images were digitally extracted for comparison in (D). The scale bars in the figure represent 1 cm in (D). Data are means ± SD (n = 10). Different letters represent a significant difference at $P < 0.05$ determined by Tukey's honest significant difference (HSD) test.

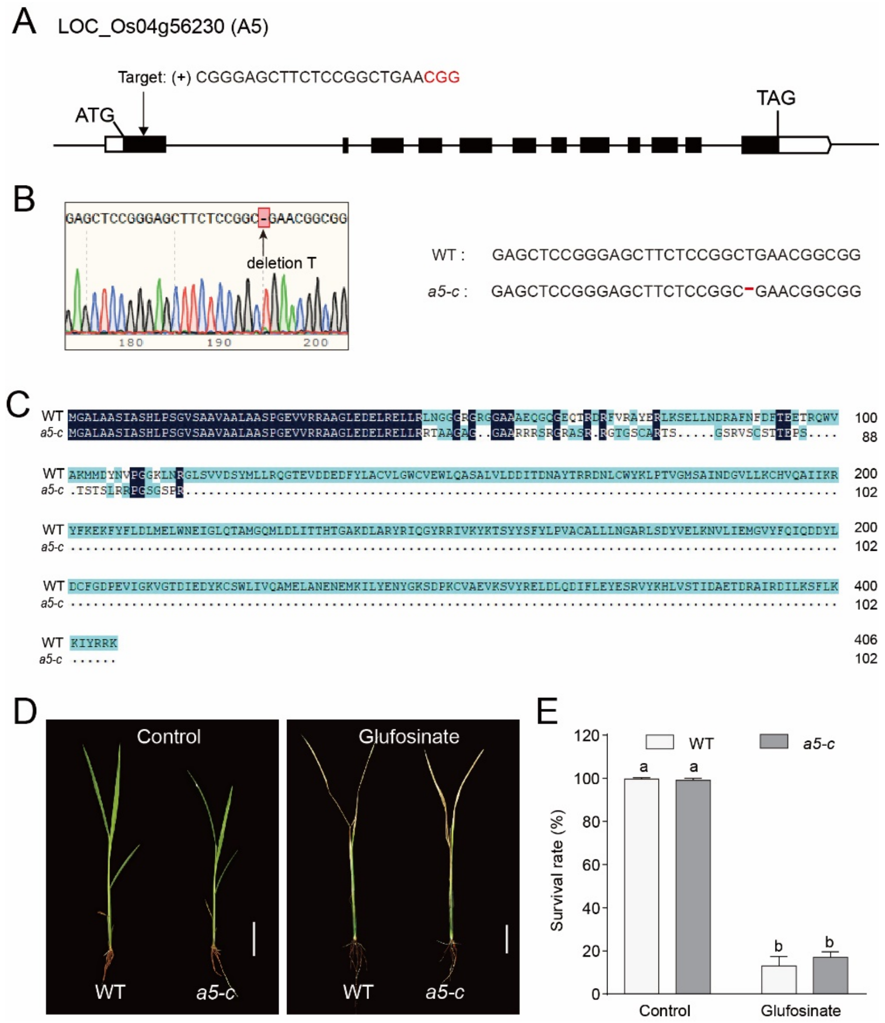

Supplementary Figure S8. Generation and analysis of LOC_Os04g56230 (A5) gene mutant using CRISPR-Cas9.

(A) Schematic representation of the LOC_Os04g56230 (A5) gene structure and the CRISPR-Cas9 target site. The target sequence and PAM are highlighted in red. (B) Sequencing chromatogram showing a single nucleotide deletion (deletion T) in the *a5-c* mutant. The WT sequence is shown for comparison. (C) Protein sequence alignment of WT and *a5-c*, showing the impact of the deletion on the amino acid sequence. (D) Phenotypic comparison of WT and *a5-c* plants under control and glufosinate-treated conditions. (E) Quantitative analysis of survival rates for WT and *a5-c* plants under control and glufosinate-treated conditions. Images were digitally extracted for comparison in (D). The scale bars in the figure represent 1 cm in (D). Data are means ± SD (n = 10). Different letters represent a significant difference at $P < 0.05$ determined by Tukey's honest significant difference (HSD) test.

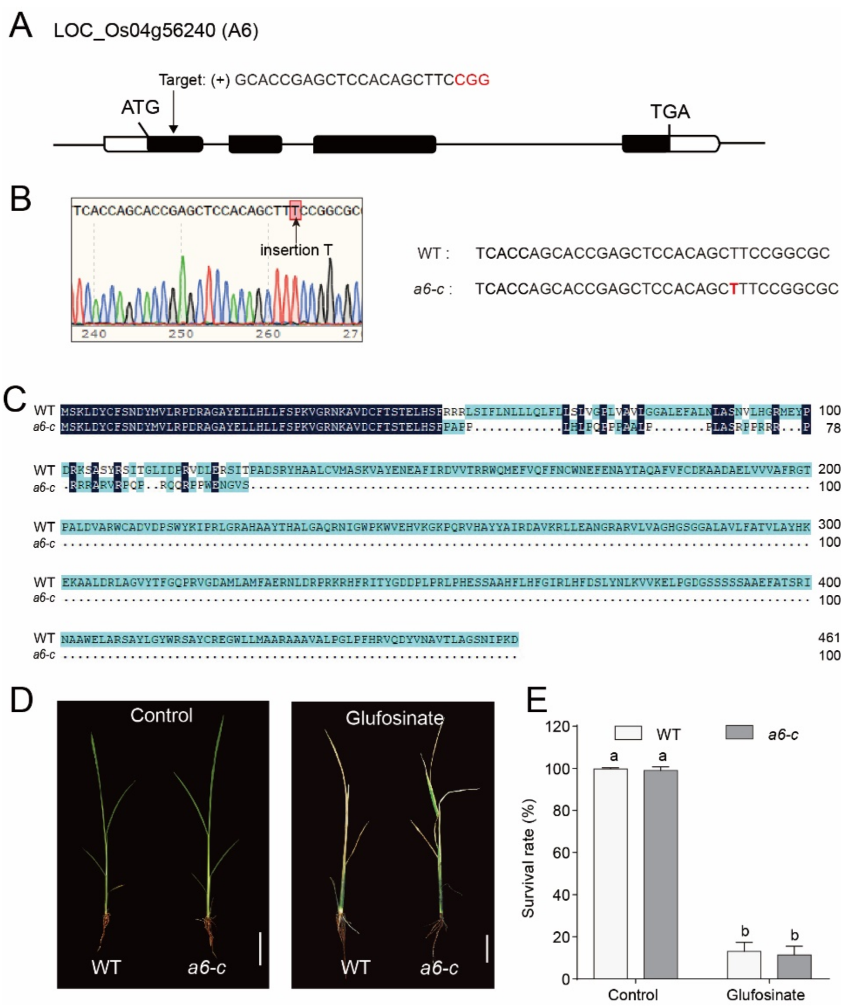

Supplementary Figure S9. Generation and analysis of LOC_Os04g56240 (A6) gene mutant using CRISPR-Cas9.

(A) Schematic representation of the LOC_Os04g56240 (A6) gene structure and the CRISPR-Cas9 target site. The target sequence and PAM are highlighted in red. (B) Sequencing chromatogram showing a single nucleotide insertion (insertion T) in the *a6-c* mutant. The WT sequence is shown for comparison. (C) Protein sequence alignment of WT and *a6-c*, showing the impact of the deletion on the amino acid sequence. (D) Phenotypic comparison of WT and *a6-c* plants under control and glufosinate-treated conditions. (E) Quantitative analysis of survival rates for WT and *a6-c* plants under control and glufosinate-treated conditions. Images were digitally extracted for comparison in (D). The scale bars in the figure represent 1 cm in (D). Data are means ± SD (n = 10). Different letters represent a significant difference at $P < 0.05$ determined by Tukey's honest significant difference (HSD) test.

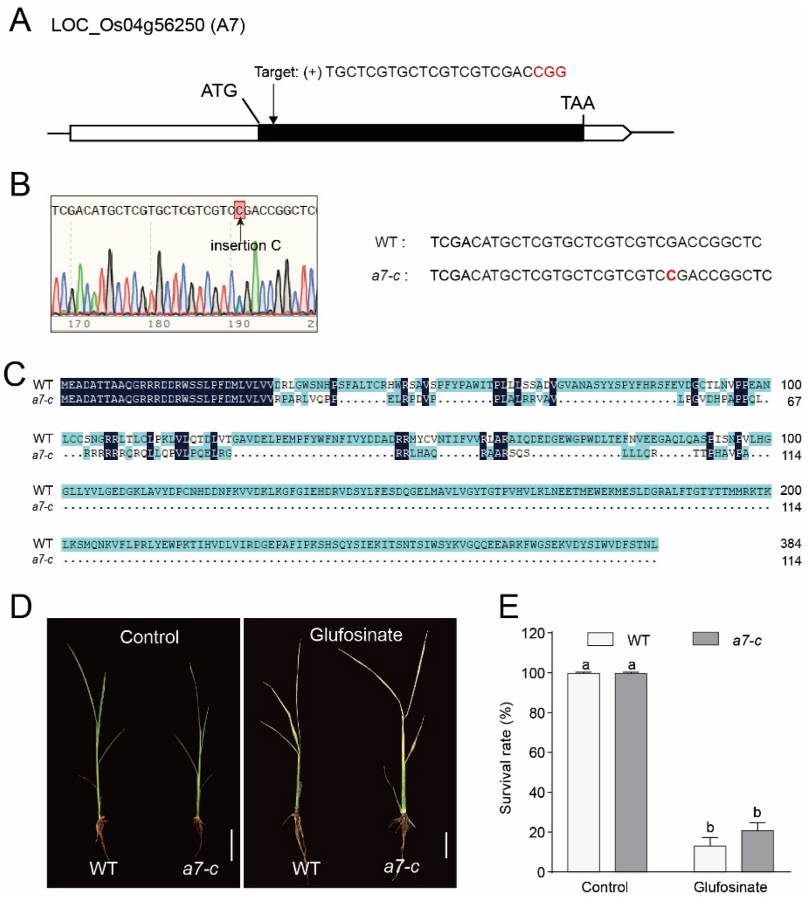

Supplementary Figure S10. Generation and analysis of LOC_Os04g56250 (A7) gene mutant using CRISPR-Cas9.

(A) Schematic representation of the LOC_Os04g56250 (A7) gene structure and the CRISPR-Cas9 target site. The target sequence and PAM are highlighted in red. (B) Sequencing chromatogram showing a single nucleotide insertion (insertion C) in the *a7-c* mutant. The WT sequence is shown for comparison. (C) Protein sequence alignment of WT and *a7-c*, showing the impact of the deletion on the amino acid sequence. (D) Phenotypic comparison of WT and *a7-c* plants under control and glufosinate-treated conditions. (E) Quantitative analysis of survival rates for WT and *a7-c* plants under control and glufosinate-treated conditions. Images were digitally extracted for comparison in (D). The scale bars in the figure represent 1 cm in (D). Data are means ± SD (n = 10). Different letters represent a significant difference at $P < 0.05$ determined by Tukey's honest significant difference (HSD) test.

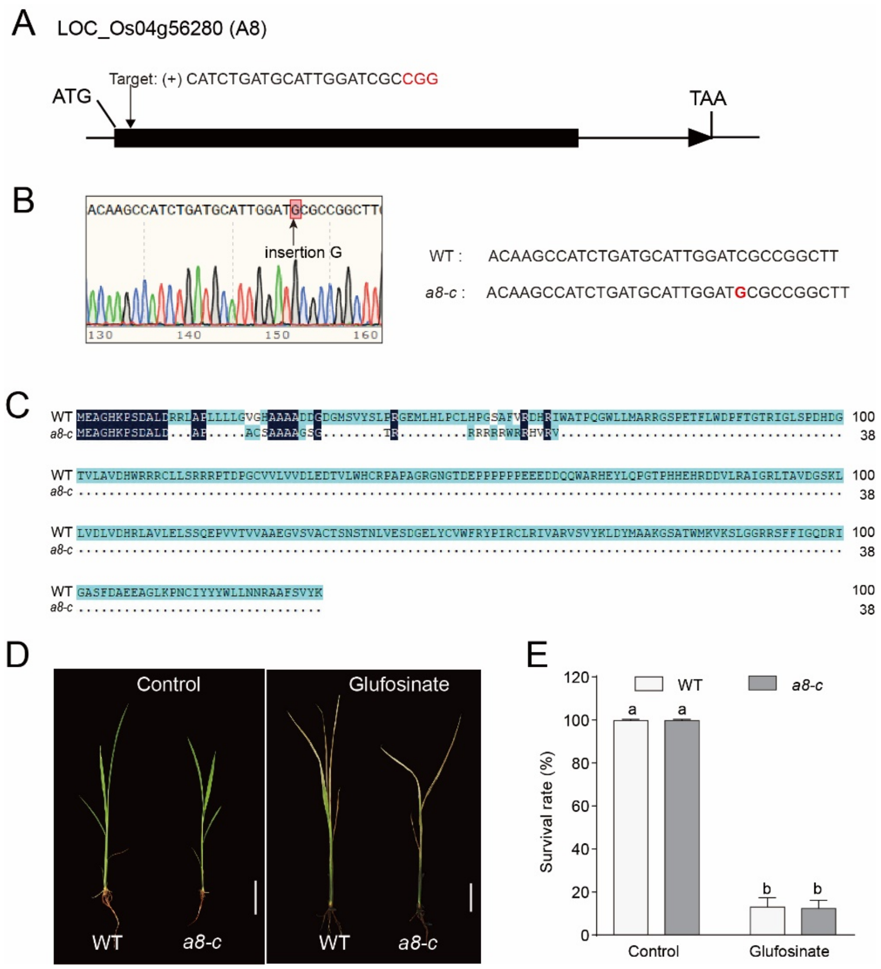

Supplementary Figure S11. Generation and analysis of LOC_Os04g56280 (A8) gene mutant using CRISPR-Cas9.

(A) Schematic representation of the LOC_Os04g56280 (A8) gene structure and the CRISPR-Cas9 target site. The target sequence and PAM are highlighted in red. (B) Sequencing chromatogram showing a single nucleotide insertion (insertion G) in the *a8-c* mutant. The WT sequence is shown for comparison. (C) Protein sequence alignment of WT and *a8-c*, showing the impact of the deletion on the amino acid sequence. (D) Phenotypic comparison of WT and *a8-c* plants under control and glufosinate-treated conditions. (E) Quantitative analysis of survival rates for WT and *a8-c* plants under control and glufosinate-treated conditions. Images were digitally extracted for comparison in (D). The scale bars in the figure represent 1 cm in (D). Data are means ± SD (n = 10). Different letters represent a significant difference at $P < 0.05$ determined by Tukey's honest significant difference (HSD) test.

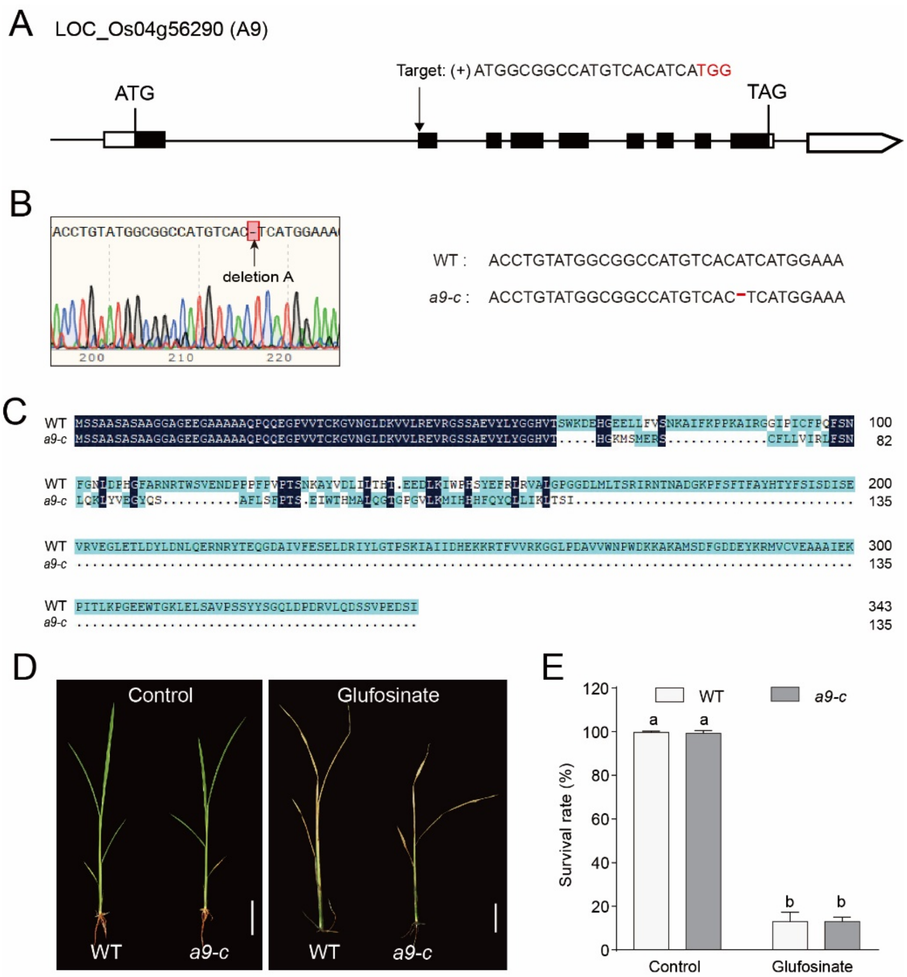

Supplementary Figure S12. Generation and analysis of LOC_Os04g56290 (A9) gene mutant using CRISPR-Cas9.

(A) Schematic representation of the LOC_Os04g56280 (A8) gene structure and the CRISPR-Cas9 target site. The target sequence and PAM are highlighted in red. (B) Sequencing chromatogram showing a single nucleotide deletion (deletion A) in the *a9-c* mutant. The WT sequence is shown for comparison. (C) Protein sequence alignment of WT and *a9-c*, showing the impact of the deletion on the amino acid sequence. (D) Phenotypic comparison of WT and *a9-c* plants under control and glufosinate-treated conditions. (E) Quantitative analysis of survival rates for WT and *a9-c* plants under control and glufosinate-treated conditions. Images were digitally extracted for comparison in (D). The scale bars in the figure represent 1 cm in (D). Data are means ± SD (n = 10). Different letters represent a significant difference at $P < 0.05$ determined by Tukey's honest significant difference (HSD) test.

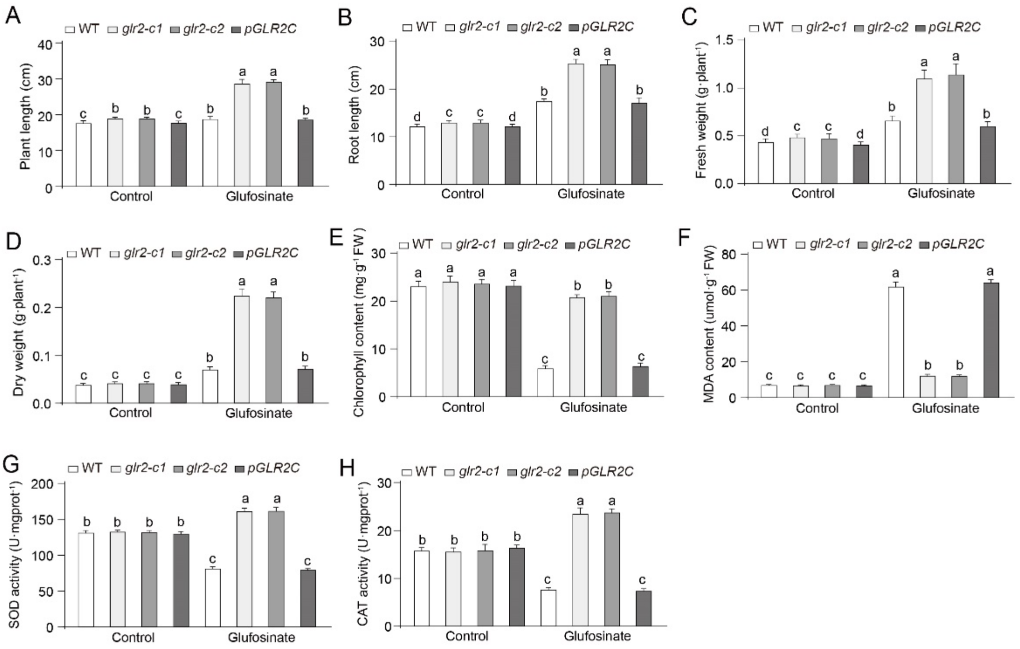

Supplementary Figure S13. Phenotypic and physiological analysis of WT, *glr2* mutant lines (*glr2-c1* and *glr2-c2*), and the *GLR2*-complemented line (*pGLR2C*) under control and glufosinate-treated conditions.

(A) Plant length, (B) Root length, (C) Fresh weight, (D) Dry weight, (E) Chlorophyll content, (F) Malondialdehyde (MDA) content, (G) Superoxide dismutase (SOD) activity, and (H) Catalase (CAT) activity were measured after treatment. The *glr2* mutant lines showed enhanced resistance to glufosinate, as indicated by superior growth metrics and lower MDA content compared to WT, while the complemented line *pGLR2C* showed similar sensitivity to glufosinate as the WT. Data are means ± SD (n = 10). Different letters represent a significant difference at $P < 0.05$ determined by Tukey's honest significant difference (HSD) test.

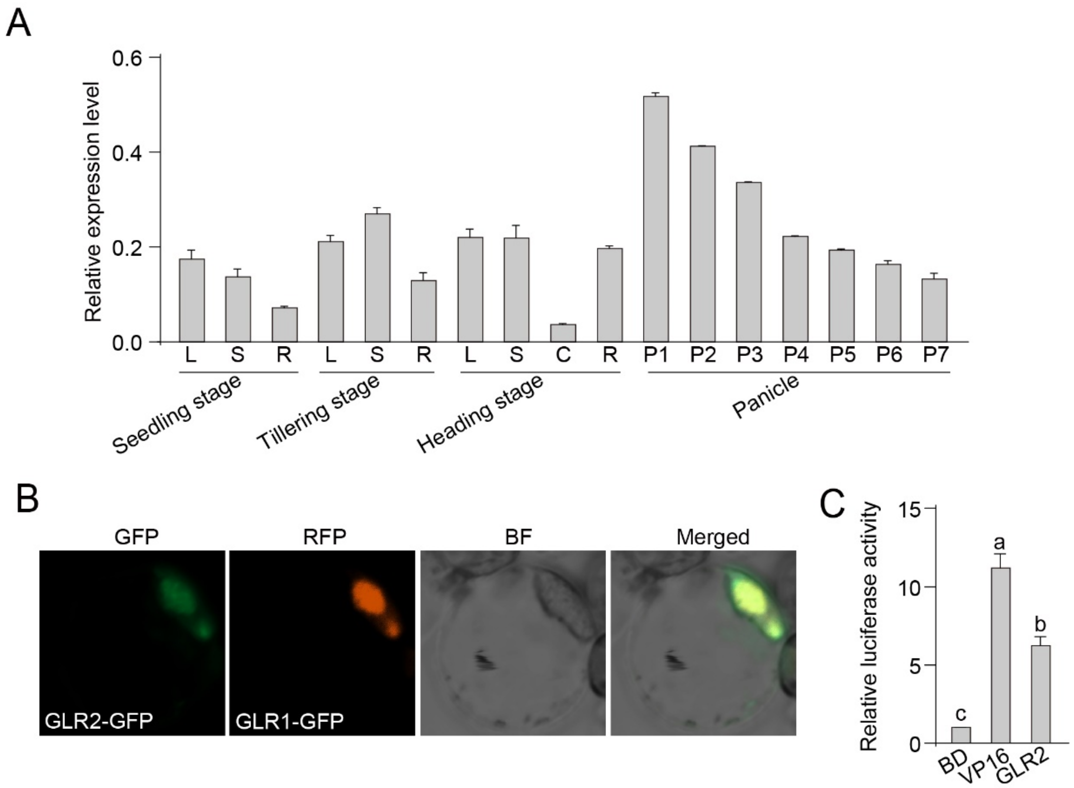

Supplementary Figure S14. Expression patterns, subcellular localization, and transcriptional activity of GLR2. (A) Relative expression levels of *GLR2* in different tissues and organs at different stage, including leaves (L), stems (S), roots (R), and panicles (P) P1=0.5 cm, P2=3 cm, P3=6 cm, P4=9 cm, P5=12 cm, P6=15 cm, P7=18 cm as indicated. (B) Subcellular localization of GLR2-GFP and GLR1-RFP fusion proteins in rice protoplasts, showing co-localization in the nucleus (merged image). BF: Bright Field. (C) Transcriptional activation assay of GLR2 in rice protoplasts, showing relative luciferase activity in different constructs: BD (empty vector control), VP16 (positive control), and GLR2. Data are means ± SD (n = 10). Different letters represent a significant difference at $P < 0.05$ determined by Tukey's honest significant difference (HSD) test.

TCTCCATAGGGGCATATTTTATTTTCTGGTTCTTTTAAATAAGATGTGCATATCTTCTTTAAGTGCTAAATTAAATTATTAACTGAAATCTAGAAAGTCATTTTAATACTCATCAGTAGCATGCAT
ACGCATAATTTCTTGATTTTATAACACTGTGGATAAGTTAATTCCTTTTTATATTGATAAATAAAACACTTTAAATTCTTGGTTGATGGATAAAGGGTGTATTTAAGATTGTGTTTTTTAGGCCCC
TTTGAATCGCATGAATGAGAAAACATAGTAATAGGAAAAACGTAGAATTTTGATAGGAATGTAAGTGTAAAACAGAGGATTACAAAACACAGGAAAAATATAGGAATGGTCGTTTGATTGGAC
CGCAGGAAAAACGCAGAAATTAGAGGAGAGATAAAGACTCAAAGGAAAGTTTCCAAGAGGTTTTAGAATTCCTCCAAAACTTATATGGCATTAGACAATCCATAAGATTTTCATAGGATTTCA
TAAGATCCATTCCTTTGATTTGAAGGGCTATATAGAAAAAATTTCTATAAAAATAAATTTCTCCAAAACTTCTATGAATTTCTTTTGAATTAAAGGTGGCCTTATTTTACGAAACAGAGGTAGTG
ATGGGAAGAGAACAAGCAACCTTCACATGCGAGTATATGCGACCGAGCTAGCTTGGCCAAGCGGTGGAAGCAGCAGCAGCAGCAGCCAGCAGCAGTGGACGCGGAAGCCTCGGCCGCG
CCGGCCACGCGAATCTCGAGGCAACGCCCGGTCGCCTCTCGCGACCTCGCCTCCCCGACCAAAACATCTCCCCGATCCCATCCCATCCCGTCCCATCGCATCATCCTCCTCCTAAACCCA
CACGTGGCAGTGCCCGCTGCCCGACGAGGTGTCCCTCCCTCGCTCACACACTTGCCCTTCGCGATTTTTCATACCAAAAAGATTTCATATAATAACTTACTAGTAACCTGATTCTGAGAAGA
ATCAAAGAAAGAATTATGAGTTTGTTTGAACCCCTTGTGAAAGAAATCACGAGATAGCGATGAGAGTGATCATGTAAATCCAGTGATCATGGAAAGAGGTCATGCAGTTTTGGTTAACACAAA
TATTTTAATTCAGTTTATTTGCAGGGCGGCTGCATGCATTTGTTTATCTCTACTTTCCAAACAAGAGAGGTGAATAATTGTGTAATCATCGGCTAATACCCCTTGTGCAAAAATATCAGGTTGAT
-766 -679 -657
CGAGTACTTGAGGAGGAGAGAGGGGTTGAACTGAAAAGTAGTGTTAATTAATCAACGAGACCACTAAACAAGAGGAGTTAACAGTGGACTGTACTAGTGGGTAGTAGTAGAAGTACTTGCT
TAAGCTCCTCTTCTTCCCTTGACTCCTCTTCTCCTCTCTTTCTCTCTCCTTTCCCCTCTCTCTCTCTCTCTATTTAGTTCTTTTGCCCCCATGTCAGCTGCAGTCTGGGGGAATCTGAGGCTGC
TGCTCACCTGAGAGACTGAAAGGAGGCAGAAGCAGCAGCAGCATCAGCATCAGCAGCAGAGCAGATAGCTGAGCTGCAGAGGGGGAGGAGAGAAGATAGTAGTGGTAGTAGTAGCTGAG
CTGTGTCAAGATTGCCCTTCAAATTCATCATCTTTTGCTCTTGTGAGAAAAGGTGAGTAGTGGTTTCTGGGTTGCGTCTCCCTTTTTCTGCAAGATTTCTGAGCCAAAATTCTCTCTTTTGCTC
GCTTTGTTCCCTTTTCTTTCTTTTCTTTTTTTCCCTTCTTCCTCCTTTCTGCTCTGCTGGTTGGTTGGTTCTTTCTCCTCCCCCAGCCTTTTTCAGCTATGAGCTCTGATTGATGATGACGATGA
TGGCGGCTGTGCAGCTGAAAAGCTTCTTCACCTTGTGGTAGTGGTAAAAGCTGTGGGAAATTTCATCCATAAAGATCGCCGTGGCGCTCGAATTCCGCTGAATTTTGTTTGTGCTCGAGCTG
ATCGGTGGAGGTGAAAGATTTTTGCGTCGAAGAG

Supplementary Figure S15. Sequence analysis of the promoter region of the *GLR1* gene, highlighting SPL binding elements. The sequence shown corresponds to the upstream region of the *GLR1* gene, with SPL binding motifs (GTAC) highlighted in red. The numbers indicate the positions of the motifs relative to the transcription start site.

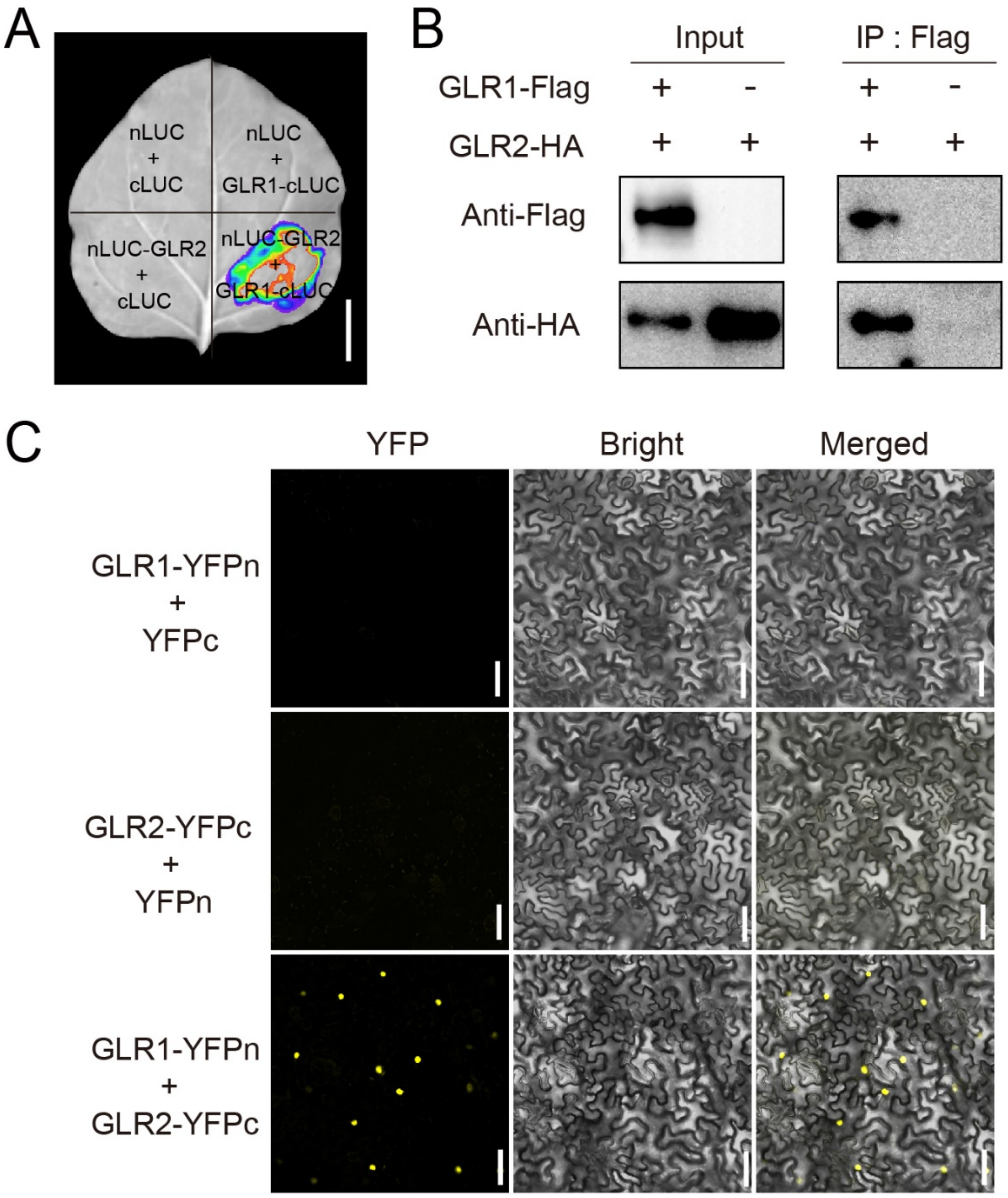

Supplementary Figure S16. Interaction between GLR1 and GLR2 proteins. (A) Luciferase Complementation Imaging (LCI) assay showing the interaction between GLR1 and GLR2 in Nicotiana benthamiana leaves. Luminescence indicates a positive interaction between GLR1-nLUC and GLR2-cLUC constructs. (B) Co-immunoprecipitation (Co-IP) assay demonstrating the physical interaction between GLR1 and GLR2 proteins. Proteins were immunoprecipitated with anti-Flag antibody and detected with anti-HA antibody, confirming the interaction. (C) Bimolecular Fluorescence Complementation (BiFC) assay showing the interaction between GLR1-YFPn and GLR2-YFPc in *Nicotiana benthamiana* epidermal cells. The reconstituted YFP fluorescence in the nucleus indicates the interaction between GLR1 and GLR2. YFPn and YFPc alone were used as controls. Scale bars = 20 µm.

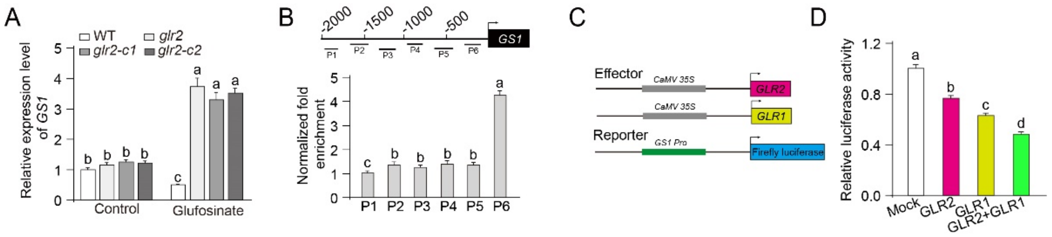

Supplementary Figure S17. Regulation of *GS1* expression by GLR1 and GLR2 in rice. (A) Relative expression levels of *GS1* in wild type (WT), *glr2*, *glr2-c1* and *glr2-c2* plants under control and glufosinate-treated conditions. (B) Chromatin immunoprecipitation (ChIP) analysis showing GLR2 binding to the *GS1* promoter. The highest enrichment was observed in region P6, located near the transcription start site. (C) Schematic representation of the effector and reporter constructs used in the luciferase assay. The *GS1* promoter was fused to a firefly luciferase reporter gene, and GLR2 and GLR1 were driven by the CaMV 35S promoter. (D) Relative luciferase activity in rice protoplasts co-transformed with *GS1* Pro-luciferase reporter and effectors (GLR2, GLR1, or both). The results indicate that GLR2 and GLR1 together strongly repress *GS1* promoter activity. Data are means ± SD (n = 5). Different letters represent a significant difference at $P < 0.05$ determined by Tukey's honest significant difference (HSD) test.

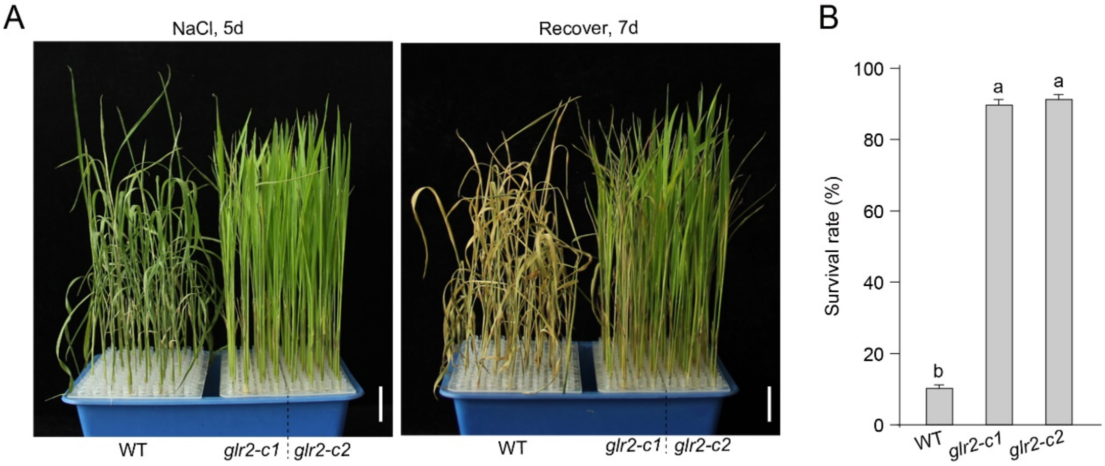

Supplementary Figure S18. Salt tolerance of *glr2-c1* and *glr2-c2* mutants compared to WT. (A) Phenotypic comparison of WT and *glr2-c1*, *glr2-c2* mutants after 5 days of NaCl treatment (150 mM) and a 7-day recovery period. The *glr2-c1* and *glr2-c2* mutants show significant recovery compared to the WT. Scale bars = 3 cm. (B) Survival rate (%) of WT, *glr2-c1*, and *glr2-c2* mutants after the 7-day recovery period. Data are presented as mean ± SD, and letters indicate statistically significant differences ($p < 0.05$).
